# Case report: a COVID-19 reactivation case

**DOI:** 10.11604/pamj.supp.2020.35.2.23326

**Published:** 2020-05-15

**Authors:** Mohamed Anass Fehdi, Anas Erragh, Amine Zerhouni, Ouissal Aissaoui, Afak Nsiri, Rachid Alharrar

**Affiliations:** 1 Department of Anesthesiology and Critical Care, University Hospital Ibn Rochd, Casablanca, Morocco

**Keywords:** COVID-19, SARS-CoV-2, ARDS, virus reactivation

## Abstract

The novel Coronavirus, named SARS-COV-2, is responsible of the COVID-19. It is a viral pneumonia that appeared in December 2019 in Wuhan, China, and is causing a pandemic. Most of patients present mild symptoms, but in many other patients, acute respiratory distress (ARDS) is more likely to be developped. The actual problematic is the appearance of cases with virus reactivation. We report a case of virus reactivation in a COVID-19 patient with ARDS.

## Introduction

The novel Coronavirus, named SARS-COV-2, is responsible of the COVID 19. It is a viral pneumonia that appeared in December 2019 in Wuhan, China, and is causing a pandemic. COVID-19 could induce symptoms including fever, dry cough, dyspnea, fatigue in patients, and might result in severe acute respiratory syndrome (SARS) and even death in severe cases. The diagnosis of COVID 19 is usually done by PCR on nasopharyngeal and throat swab samples. As of today, the curative treatments are still debated. The actual problematic is the appearance of cases with virus reactivation. We report a case of virus reactivation in a COVID 19 patient with ARDS.

## Observation

**Figure 1:**
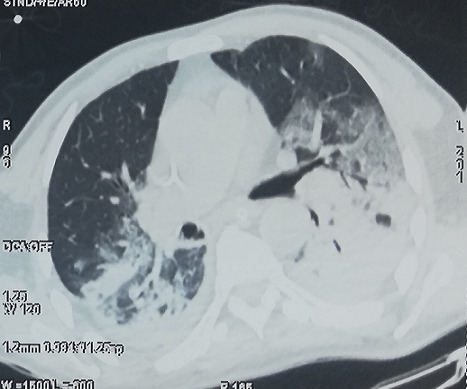
Thoracic CT-scan 1

A 69-year-old man, with no medical history, was admitted in our COVID intensive care unit for viral pneumonia on 24th March 2020. He explained that he had been traveling in an overcrowded city, that was considered later as a endemic zone of SARS-COV-2 from 14th to 20th March, and he confirmed not being in contact with no confirmed case of COVID-19. On 21 March, he suffered from fever of 38.8°C with cough and dispnea. He first had came to an affiliated hospital where a high-resolution computed tomography was performed (Figure 1), showing bilateral ground-glass opacities, with multilobe and posterior involvement, with no signs of pulmonary embolism. An rt-PCR on nasopharyngeal swab was positive, the patient was confirmed as a COVID-19 patient.

On admission, the clinical evaluation revealed a normal neurological and hemodynamic state, the respiratory rate was at 35, the oxygen saturation at 85% without oxygen, and at 94% with high-concentration mask. Arterial blood gas analysis showed respiratory alkalosis with hypoxemia, arterial oxygen tension (PaO2) at 45 mmHg. The initial conditioning included, a peripheral intravenous line (PIV), a high-concentration oxygen mask, a half-seated position (45°). Biological assessment had revealed an important inflammatory syndrome with hyperferritinemia at 3350 ng/mL, elevated CRP at 520mg/L, and lymphopenia at 180.106/uL, high fibrinogen at 7.52g/L, elevated D-Dimer at 2.4 ng/L. Liver function, renal function, myocardial enzymes, electrolyte, and serum procalcitonin were normal. Initial treatment was started with Hydroxychloroquine 200mg x 3/day, Azithromicyne 500mg/day, Céftriaxone 2g/day, and Moxifloxacin 400mg x 2/day, proton pump inhibitor, and thromboprophylaxis. 48 hours after his admission, the patient presented a respiratory deterioration.

**Figure 2:**
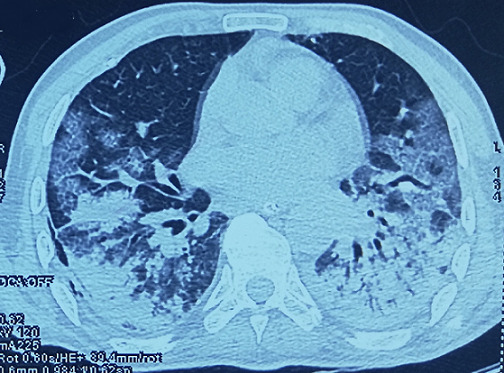
Thoracic CT-scan control

An orotracheal intubation had been performed and the patient was put under continuous sedation and mechanical ventilation for 8 days. The evolution was marked by a clinical and biological improvement, the inflammatory syndrome decreased (ferritinemia at 1050) and the lymphocyte account increased (at 1100). Weaning of mechanical ventilation was started then, and the patient had been extubated after 8 days of mechanical ventilation. The SARS-COV-2 PCR at day 14 and 15 after onset were negative. However, 3 days later, at day 18 after onset, and day 8 after extubation, the patient presented signs of respiratory deterioration, the patient was re-intubated. SARS-COV-2 PCR realized were again positive, and the biological assessment showed signs of deterioration, with an important inflammatory syndrome (hyperferritinaemia at 8500 ng/mL, elevated CRP at 480 mg/L, elevated fibrinogen at 8.42g/L), and lymphopenia at 200.106/uL. A second SARS-COV-2 PCR was positive as well. A thoracic CT-Scan was performed (Figure 2), showing worsening of radiological images. Which indicated the possible reactivation of COVID-19. The patient died 10 days later due to multi-organ failure secondary to cytokine storm.

## Discussion

Nowadays, The SARS-COV-2 is responsible of a worldwide pandemic. It is causing a large spectrum of disease, from mild cases to life-threatening. With a high risk of developing ARDS and/or multiple organ failure [1]. Patients with positive SARS-COV-2 RNA on respiratory tract specimens are considered COVID-19 positive. Therefore they are an infectious source, whether they are symptomatic or not [1]. The virus reactivation or reinfection is still poorly investigated. Most of the cases reported in litterature, are asymptomatic, and have been detected during routine tests [1,2]. No case of ARDS patient has been reported [2]. We believe that our patient is a first case of possible reactivation in ARDS case. In favour of our assumption are the clinical and biological detetrioration that might be due to a reactivation of the viral process and the cytokine storm.

We can not exclude the possibility of false negative SARS-COV-2 PCR, in fact the result of the SARS-CoV-2 RNA test depends on the viral charge in the specimen [1]. Therefore, it is possible to have false negatives for oropharyngeal or nasopharyngeal swab tests. It could be explained by many factors, such as, the site of test, the operator´s experience, and the viral load. The Bronchoalveolar lavage fluid (BALF) specimen, although it has a high exposure risk, it is considered accurate. Moreover, SARS-CoV-2 RNA can be found in patient´s sputum, blood, or stool swab by RT-PCR. In order to maximize sensitivity it is more effective to multiply tests [1,2]. Finally, we recommand a larger cohort in order to investigate more the characteristics of the COVID-19 reactivation.

## Conclusion

Covid 19 reactivation is a new concept and a real challenge for practitioners. It does need more investigation in order to improve our patients management.

## Competing interests

The author declares no competing interests.
